# Genotyping and phenotyping strategies for genetic improvement of meat quality and carcass composition in swine

**DOI:** 10.1186/s12711-022-00736-4

**Published:** 2022-06-07

**Authors:** Emmanuel André Lozada-Soto, Daniela Lourenco, Christian Maltecca, Justin Fix, Clint Schwab, Caleb Shull, Francesco Tiezzi

**Affiliations:** 1 Department of Animal Science, North Carolina State University, Raleigh, NC 27695 USA; 2 Department of Animal and Dairy Science, University of Georgia, Athens, GA 30602 USA; 3 Acuity Ag Solutions, LLC, Carlyle, IL 62230 USA; 4 The Maschhoffs, LLC, Carlyle, IL 62230 USA; 5 Department of Agriculture, Food, Environment and Forestry (DAGRI), University of Florence, 50144 Florence, Italy

## Abstract

**Background:**

Meat quality and composition traits have become valuable in modern pork production; however, genetic improvement has been slow due to high phenotyping costs. Combining genomic information with multi-trait indirect selection based on cheaper indicator traits is an alternative for continued cost-effective genetic improvement.

**Methods:**

Data from an ongoing breeding program were used in this study. Phenotypic and genomic information was collected on three-way crossbred and purebred Duroc animals belonging to 28 half-sib families. We applied different methods to assess the value of using purebred and crossbred information (both genomic and phenotypic) to predict expensive-to-record traits measured on crossbred individuals. Estimation of multi-trait variance components set the basis for comparing the different scenarios, together with a fourfold cross-validation approach to validate the phenotyping schemes under four genotyping strategies.

**Results:**

The benefit of including genomic information for multi-trait prediction depended on the breeding goal trait, the indicator traits included, and the source of genomic information. While some traits benefitted significantly from genotyping crossbreds (e.g., loin intramuscular fat content, backfat depth, and belly weight), multi-trait prediction was advantageous for some traits even in the absence of genomic information (e.g., loin muscle weight, subjective color, and subjective firmness).

**Conclusions:**

Our results show the value of using different sources of phenotypic and genomic information. For most of the traits studied, including crossbred genomic information was more beneficial than performing multi-trait prediction. Thus, we recommend including crossbred individuals in the reference population when these are phenotyped for the breeding objective.

## Background

Meat quality and carcass composition in commercial animals are becoming traits of high interest and economic value in the pork industry. Increased consumer preference for pork with high nutritional and appealing organoleptic characteristics [1, 2] and the producers’ demand for a higher yield of valuable primal cuts have intensified the need for continued genetic improvement of carcass traits. In swine, selection has been traditionally performed within purebred nucleus lines, whereas crossbreeding is used to produce commercial animals to take advantage of breed complementarity and heterosis. Ideally, to achieve sufficient genetic gain for crossbred performance in such a breeding scheme, a large number of phenotypic records for the traits of interest measured on crossbred offspring of the selection candidates should be collected, in addition to keeping accurate pedigree information. Unfortunately, selecting for carcass traits in swine is hindered by the inability to record carcass trait phenotypes on selection candidates, since these phenotypes must often be measured post-mortem, and by the high cost of recording these post-mortem phenotypes, which often need expensive wet-lab analyses (e.g., muscle lipid ethereal extract for the determination of intramuscular fat). For all these reasons, direct selection for these traits is economically unsustainable. Thus, the use of traits that can be recorded at a lower cost for indirect selection is appealing but has not been widely investigated.

The most straightforward approach to predict crossbred performance for traits of economic interest is to use purebred performance for cheap and routinely measured growth traits. For example, intramuscular fat can be measured post-mortem with wet-lab analyses or with an ultrasound probe on the live animal, with the latter being a predictor of the former. The correlated response that can be achieved for crossbred gain using purebred performance depends largely on the genetic correlation between crossbred and purebred performance. This might be affected by differences in the rearing environment, the genetic distance between the breeds used for crossbreeding, and differences in genetic architecture and trait definitions for purebreds versus crossbreds [3, 4]. Several studies have found that the heritabilities of crossbred and purebred performance differ significantly and that the genetic correlation between purebred and crossbred performance for the same trait is less than 1 in swine [5–8], broilers [9], and beef cattle [10].

Another possible approach is to use crossbred performance for growth and routinely measured carcass traits for indirect selection of crossbred meat quality. Recent studies have found moderate to high genetic correlations of meat quality and carcass traits with growth and performance in crossbred animals [11, 12], which often meet the genetic correlation threshold of 0.5, which is considered as the requirement to make valuable use of correlated traits in multiple-trait models [13]. However, in reality, these traits are rarely measured on the same animals and results on carrying information between purebred and crossbred swine populations have been mixed [14].

In the era of genomic selection, genetic gain for crossbred performance depends not only on the phenotyping strategy but also on the genotyping strategy used. While simulation studies have shown that genomic selection can increase accuracy of selection indices for crossbred performance, its effect will depend on marker density, size of the reference population, and the relationship between training and validation populations [15–19]. In practice, potential genotyping strategies can include genotyping the purebred selection candidates, the crossbred dams of commercial animals, the crossbred offspring on which the traits are measured, or any combination of these. The ultimate choice of genotyping strategy depends on costs, logistics, breeding goals, and the gain in prediction accuracy per genotyped animal. In reality, the usefulness of purebred or crossbred genotypic and phenotypic data for prediction of breeding values varies. When the correlation between purebred and crossbred performance is high and the relationship between the crossbred reference population and purebred selection candidates is low, inclusion of crossbred phenotypes have been shown to not provide further accuracy in breeding value prediction [15, 20, 21]. In a recent simulation study, See et al. [22] showed that the inclusion of crossbred phenotypic and genotypic information increased selection response for crossbred performance most when purebred–crossbred correlations were low.

The availability of phenotypic and genomic information on crossbreds allows the evaluation of different genotyping and phenotyping strategies in terms of selection efficiency for commercial traits of interest. Therefore, the aim of this study was to evaluate the impact of single and multiple-trait models with different phenotyping and genotyping strategies to predict crossbred meat and carcass quality traits.

## Methods

### Data structure and genomic information

The animals used in this study included commercial crossbred (CB) and purebred Duroc (PB) pigs from 28 paternal half-sib families raised at The Maschhoffs LLC (now Acuity Ag Solutions, Carlyle, IL, USA). Crossbred pigs were from a three-way cross that involved 28 purebred Duroc boars and crossbred Yorkshire $\times$ Landrace or Landrace $\times$ Yorkshire sows. The purebred Duroc pigs were offspring of the same 28 Duroc sires. Matings that produced the crossbred and purebred pigs were carried out from November to December 2014 and data collection was from March to November 2015. The purebred individuals were kept in a facility free of the porcine respiratory and reproductive syndrome virus (PRRS) and the porcine epidemic diarrhea (PED), while no information was available about the facility that raised the crossbred individuals. Both populations were fed the same diet. Additional information on the rearing conditions of purebred and crossbred individuals is in Maltecca et al. [23]. Phenotypic data were collected for 1171 to 5294 CB and 1681 to 3106 PB individuals, depending on the trait. Additional information on the collection of phenotypic data is in Khanal et al. [11] and Bergamaschi et al. [6]. Genomic information on the 28 PB Duroc sires, the 914 dams of CB pigs, 1252 CB and 1200 PB Duroc pigs was obtained using the porcine single nucleotide polymorphism (SNP)60 v2 BeadChip (Illumina, Inc.). After tracing five generations back, the pedigree for the combined CB and PB population included 96,234 animals. Standard quality control procedures were performed on the combined genomic data for PB and CB individuals, which removed non-autosomal SNPs and SNPs with a call rate lower than 0.90 and/or minor allele frequency lower than 0.05. In total, 42,529 SNPs remained after quality control.

### Traits measured on crossbred pigs

Traits recorded on the CB progeny included the meat quality traits of intramuscular fat (cIMF), Warner–Bratzler slice shear force (cSSF), Minolta L* (cM.L), Minolta a* (cM.a), Minolta b* (cM.b), pH (cPH), subjective color (cSCOL), subjective firmness (cSFIR), and subjective marbling (cSMAR); the carcass composition traits of loin weight (cLOI), belly weight (cBEL), ham weight (cHAM), loin depth (cCLD), backfat depth (cCBF), and carcass average daily gain (cCDG); and the growth traits measured on live animals at market weight of live loin depth (cLLD), live backfat (cLBF), and live average daily gain (cLDG). Growth and some carcass traits (cLLD, cLBF, cLDG, cCLD, cCBF, cCDG) were recorded on all the individuals, but the meat quality and carcass dissection traits (cIMF, cSSF, cM.L, cM.a, cM.b, cPH, cSCOL, cSFIR, cSMAR, cLOI, cBEL, cHAM) were recorded on a selection of individuals that were chosen in equal numbers from each ‘group’, defined as the paternal half-sib individuals of the same sex and housed in the same pen. Four to five individuals were chosen for each group: the individual closest to the group’s mean for carcass growth, and the individuals closest to the + 1 standard deviation, + 2 standard deviations, − 1 standard deviation, and − 2 standard deviations from this mean. This selection effectively covered the whole within-group distribution and can be viewed as a viable routine phenotyping strategy. The individuals that were chosen for additional phenotyping were also chosen for genotyping.

Both cCBF and cCLD were measured after slaughter using a Fat-O-Meater probe (SFK Technology A/S, Herlev, Denmark) near the 10th rib. cCDG was calculated by dividing the difference between the hot carcass weight and birth weight by the pig's age at slaughter. Carcasses were split, blast-chilled for 90 min, and then separated into primal cuts, such that cLOI, cBEL, and cHAM could be measured. Separation and preparation of the loins to measure meat quality traits were done as described by Wilson et al. [24]. cPH was measured on the ventral side of the *longissimus dorsi* muscle using a handheld MPI pH meter fitted with a glass electrode (Meat Probes Inc., Topeka, KS). Thirty minutes post-slicing (during which oxygenation of the myoglobin occurs), the instrumental color traits were measured using a Minolta CR-400 Chroma meter (Minolta Camera Co., Ltd., Osaka, Japan), including cM.L (luminosity), cM.a (redness), and cM.b (yellowness). Subjective measures of pork quality were recorded at the same time, including cSCOL (5 categories), cSFIRM (on a 1 to 5 scale) and cSMAR (6 categories). Further details on the subjective color and marbling categories are in Wilson et al. [24] and on subjective firmness are in Khanal et al. [11]. Measurement of cSSF and cIMF were determined as described by Wilson et al. [24].

### Traits measured on purebred pigs

Traits recorded on purebred animals included intramuscular fat (pIMF), backfat (pLBF), loin depth (pLLD), loin eye area (pLLA), nursery average daily gain (pLDG1), and finisher average daily gain (pLDG2). Purebred Duroc pigs were kept in a facility free of the porcine reproductive and respiratory syndrome and porcine epidemic diarrhea. After weaning, the pigs were moved to nursery conditions on another site, after which the pigs entered a mid-test phase and were allocated to single-gender pens. At this point, pLDG1 was calculated by dividing the difference between mid-test weight and weaning weight by the number of days spent in the nursery. When the pigs were off-tested at 180 days, pIMF, pLBF, pLLD, and pLLA were measured using an ultrasound probe (Biotronics Inc., Ames, IA, USA). The trait pLDG1 was calculated by dividing the difference between the pig's off-test weight and mid-test weight by the number of days spent in finishing [6]. The number of records and descriptive statistics for each trait are in Table 1.

**Table 1:** Descriptive statistics and heritability estimates for all traits using single-trait models

Trait	Definition	N	Mean	Median	SD	Min	Max	Heritability
Crossbred								
cIMF (%)	Intramuscular fat (Ethereal extract)	1227	2.71	2.61	0.93	0.44	7.23	0.35 (0.14, 0.58)
cSSF (kg)	Slice-shear force (Warner–Bratzler)	1227	15.93	15.30	3.65	9.06	39.93	0.19 (0.07, 0.33)
cM.L	Luminosity (Minolta camera)	1241	45.3	45.17	3.10	35.98	56.58	0.11 (0.01, 0.23)
cM.a	Redness (Minolta camera)	1241	3.79	3.72	1.10	0.68	7.89	0.14 (0.03, 0.25)
cM.b	Yellowness (Minolta camera)	1241	− 0.15	− 0.22	0.90	− 2.45	3.43	0.04 (0.00, 0.12)
cPH	pH	1171	5.64	5.62	0.18	5.03	6.91	0.06 (0.00, 0.17)
cSCOL	Color (subjective)	1237	2.72	2.50	0.47	1.50	4.00	0.22 (0.08, 0.36)
cSFIR	Firmness (subjective)	1237	3.04	3.00	0.99	1.00	5.00	0.04 (0.00, 0.12)
cSMAR	Marbling (subjective)	1237	3.10	3.00	0.83	1.00	6.00	0.11 (0.00, 0.24)
cCLD (mm)	Loin depth (Fat-O-Meater)	4894	66.78	67.00	6.91	36.00	89.00	0.05 (0.01, 0.10)
cCBF (mm)	Backfat depth (Fat-O-Meater)	4893	22.62	22.00	4.88	10.00	51.00	0.28 (0.13, 0.44)
cCDG (kg)	Carcass average daily gain	5117	0.52	0.52	0.06	0.30	0.89	0.05 (0.01, 0.09)
cLLD (mm)	Loin depth (Ultrasound)	5294	60.28	60.20	5.14	39.12	80.52	0.13 (0.05, 0.22)
cLBF (mm)	Backfat depth (ultrasound)	5294	23.51	22.86	6.38	8.38	54.61	0.23 (0.11, 0.37)
cLDG (kg)	Live daily gain	5289	0.69	0.69	0.08	0.42	0.95	0.05 (0.00, 0.11)
cLOI (kg)	Loin weight	1255	23.09	22.97	2.17	16.93	31.01	0.06 (0.00, 0.17)
cBEL (kg)	Belly weight	1251	40.31	40.20	6.12	23.60	58.9	0.04 (0.00, 0.11)
cHAM (kg)	Ham weight	1255	55.53	55.59	5.17	39.90	70.70	0.06 (0.00, 0.15)
Purebred								
pIMF (%)	Intramuscular fat (ultrasound)	2701	2.49	2.50	0.83	0.10	7.60	0.11 (0.04, 0.18)
pLBF (mm)	Live backfat depth (ultrasound)	3036	16.23	15.75	3.95	5.59	34.29	0.32 (0.19, 0.48)
pLLD (cm)	Live loin depth (ultrasound)	1681	6.00	5.99	0.55	2.59	7.85	0.18 (0.03, 0.35)
pLLA (cm^2^)	Live loin area (ultrasound)	3036	50.69	50.77	6.26	26.45	71.94	0.15 (0.06, 0.26)
pLDG1 (kg)	Live daily gain, nursery	3106	0.45	0.46	0.09	0.10	0.77	0.30 (0.15, 0.45)
pLDG2 (kg)	Live daily gain, finisher	2967	0.89	0.90	0.12	0.41	1.29	0.23 (0.12, 0.36)

95% highest posterior density interval for heritability estimates are shown in parenthesis

### Indirect selection for carcass traits using purebred growth phenotypes

Traits that are routinely measured in the purebred nucleus line, such as growth traits, could serve as a cheap alternative for improving carcass phenotypes in crossbreds. Using purebred growth measures, we constructed several selection scenarios to quantify their relevance in the selection for crossbred carcass traits (see Table 2). We ordered scenarios based on the cost of phenotype collection. In the first scenario (PB-1), we included only PB average daily gain traits (pLDG1 and pLDG2) among the predictors since these traits require only weight recording. In the second purebred trait scenario (PB-2), we expanded the set of predictors to include PB ultrasound traits (pLBF, pLLD, and pLLA), along with the traits from scenario PB-1. In the third purebred trait scenario (PB-3), we included pIMF in addition to the traits from scenario PB-2. The PB-3 scenario was evaluated because recording pIMF currently requires additional software costs compared to the other PB ultrasound traits.

**Table 2:** Traits in the breeding objective and in the selection criterion for the evaluated phenotyping and breeding objective scenarios

Phenotyping scenario	Selection criterion (Traits)	Breeding objective (Trait)
ST	Same as breeding objective	cIMF, cSSF, cSMAR, cSFIR, cSCOL, cM.L, cM.a, cM.b, cPH, cLOI, cCDG, cCBF, cCLD, cBEL, or cHAM
PB-1	pLDG1 and pLDG2	
PB-2	pLDG1, pLDG2, pLBF, pLLD and pLLA	
PB-3	pLDG1, pLDG2, pLBF, pLLD, pLLA and pIMF	
CB-Live	cLLD, cLBF, and cLDG	cIMF, cSSF, cLOI, cSMAR, cSFIR, cSCOL, or cLOI
CB-FOM	cCBF, cCLD and cCDG	
CB-Color	cM.L, cM.a, cM.b and cPH	

Trait definitions are in Table 1

### Less expensive crossbred phenotypes for the selection of meat quality

To overcome the hurdles of genomic prediction between purebred and crossbred pigs, we investigated the possibility to predict high-value CB meat quality traits in crossbreds using models that include cheaper-to-measure CB traits. For this purpose, three crossbred trait scenarios (see Table 2) that included measurement of different sets of ultrasound growth traits on CB, depending on the cost and ease of recording, were evaluated. The first crossbred scenario (CB-Live) included only live growth traits measured on crossbreds (cLLD, cLBF, and cLDG) using a weighing scale and ultrasound probe, the second crossbred trait scenario (CB-FOM) included traits measured on the carcass using the Fat-O-Meater probe (cCBF and cCLD) and carcass average daily gain (cCDG) and the third crossbred trait scenario (CB-Color) included traits that require carcass dissection, such as color (cM.L, cM.a, and cM.b) and pH (cPH). For each of these scenarios, we estimated variance components as well as predictive ability of models for the meat quality traits of cIMF, cSSF, cSCOL, cSFIR, and cSMAR, and for cLOI as the sole carcass composition trait. These six traits were selected because of their relatively higher phenotyping cost, which prevents their use as predictors.

### Statistical analyses

Variance components were estimated using pedigree information for each of the seven selection scenarios: ST, PB-1, PB-2, PB-3, CB-Live, CB-FOM, and CB-Color. The model for the CB traits was:

1 $$\mathbf{y}=\mathbf{X}{\varvec{\upbeta}}+{\mathbf{Z}}_{\mathbf{l}}\mathbf{l}+{\mathbf{Z}}_{\mathbf{p}}\mathbf{p}+{\mathbf{Z}}_{\mathbf{a}}\mathbf{a}+\mathbf{e},$$

where $\mathbf{y}$ is the vector of phenotypes for the investigated trait; ${\varvec{\upbeta}}$ is the vector of solutions for fixed effects, including dam line, gender, and contemporary group; $\mathbf{l}$ is the vector of solutions for the random effect of litter, with $\mathbf{l}\sim N(\mathbf{0},\mathbf{I}{\upsigma }_{\mathrm{l}}^{2})$, where $\mathbf{I}$ is an identity matrix and ${\upsigma }_{\mathrm{l}}^{2}$ is the estimated litter variance; $\mathbf{p}$ is the vector of solutions for the random effect of the pen, with $\mathbf{p}\sim N(\mathbf{0},\mathbf{I}{\upsigma }_{\mathrm{p}}^{2})$ where ${\upsigma }_{\mathrm{p}}^{2}$ is the estimated pen variance; $\mathbf{a}$ is the vector of solutions for the random additive genetic effect of the animal, with $\mathbf{a}\sim N(\mathbf{0},\mathbf{A}{\upsigma }_{\mathrm{a}}^{2})$, where $\mathbf{A}$ is the numerator relationship matrix built on a pedigree traced nine generations back and ${\upsigma }_{\mathrm{a}}^{2}$ is the estimated additive genetic variance; $\mathbf{X}$ is the incidence matrix of fixed effects; ${\mathbf{Z}}_{\mathbf{l}}$, ${\mathbf{Z}}_{\mathbf{p}}$, and ${\mathbf{Z}}_{\mathbf{a}}$ are the corresponding incidence matrices for the random effects; and $\mathbf{e}$ is the vector of random residuals, with $\mathbf{e}\sim N(\mathbf{0},\mathbf{I}{\upsigma }_{\mathrm{e}}^{2})$, where ${\upsigma }_{\mathrm{e}}^{2}$ is the residual variance. In the pedigree, animals in the base population were considered unrelated and unknown parent groups were not used. As compared to previous work on the same data, the model used was partially different because we were able to incorporate further information in the dataset. In particular, in this work we were able to add a litter permanent environmental effect, which was not used in Khanal et al. [11].

The model for the PB traits was:

2 $$\mathbf{y}= \mathbf{X}{\varvec{\upbeta}} + {\mathbf{Z}}_{\mathbf{l}}\mathbf{l} + {\mathbf{Z}}_{\mathbf{a}}\mathbf{a} +\mathbf{e},$$

where the fixed effects included contemporary group, parity of the birth sow, and age at recording (age at on-test for pLDG1 and age at off-test for other PB traits), and the other terms are as defined in Model (1).

Single-trait models were used for the ST scenario and multiple-trait models for the other scenarios. Models were implemented in the GIBBS3F90 program (v. 1.83) from the BLUPF90 family of programs [25, 26]. The single-trait models were run for 100,000 iterations, with the first 20,000 samples discarded and samples saved every 20th iteration, leaving a total of 4000 samples for subsequent inference. The multiple trait models were run for 600,000 iterations, with the first 200,000 samples discarded and every 20th sample saved, leaving a total of 20,000 samples for subsequent inference. Convergence was assessed by visual inspection of the trace plots.

### Prediction of crossbred traits

We used fourfold cross-validation based on k-means clustering to compare the different scenarios for their ability to predict unobserved phenotypes in CB individuals. Dissimilarities between the 28 purebred sires were derived from the pedigree relationship matrix and were used to allocate individuals to clusters, maximizing intra-group and minimizing inter-group additive genetic relationships [27, 28].

For the ST scenario, breeding values were estimated by masking phenotypes for one of the four groups and prediction accuracy was calculated as the correlation between the estimated breeding values and the (masked) phenotypes adjusted for the fixed effects (see Model 1). The solutions for the fixed effects were calculated on the full dataset. This allowed the prediction of the (adjusted) phenotype for the CB individuals belonging to a group that was not yet tested (e.g., group 4) based on information from the other groups (e.g., groups 1–2–3).

For the PB-1, PB-2, and PB-3 multi-trait prediction scenarios, the grouping was maintained for the CB trait to be predicted, but PB phenotypic information from all groups was included in the model. This mimicked a testing system where PB information is collected on all groups, but CB information was masked for the validation group. In other words, it was assumed that PB phenotypic information is available for the paternal half-sib families included in the validation set but not CB information (assumed as the breeding goal). For the CB-Live, CB-FOM, and CB-Color scenarios, the phenotypic information for the predictor traits was included for all groups and the phenotypic information for the trait to be predicted (e.g., cSSF) was masked for the validation group. For example, cLBF is recorded on groups 1–2–3–4, but cSSF (assumed as the breeding goal) is recorded only on groups 1–2–3.

**Fig. 1:**
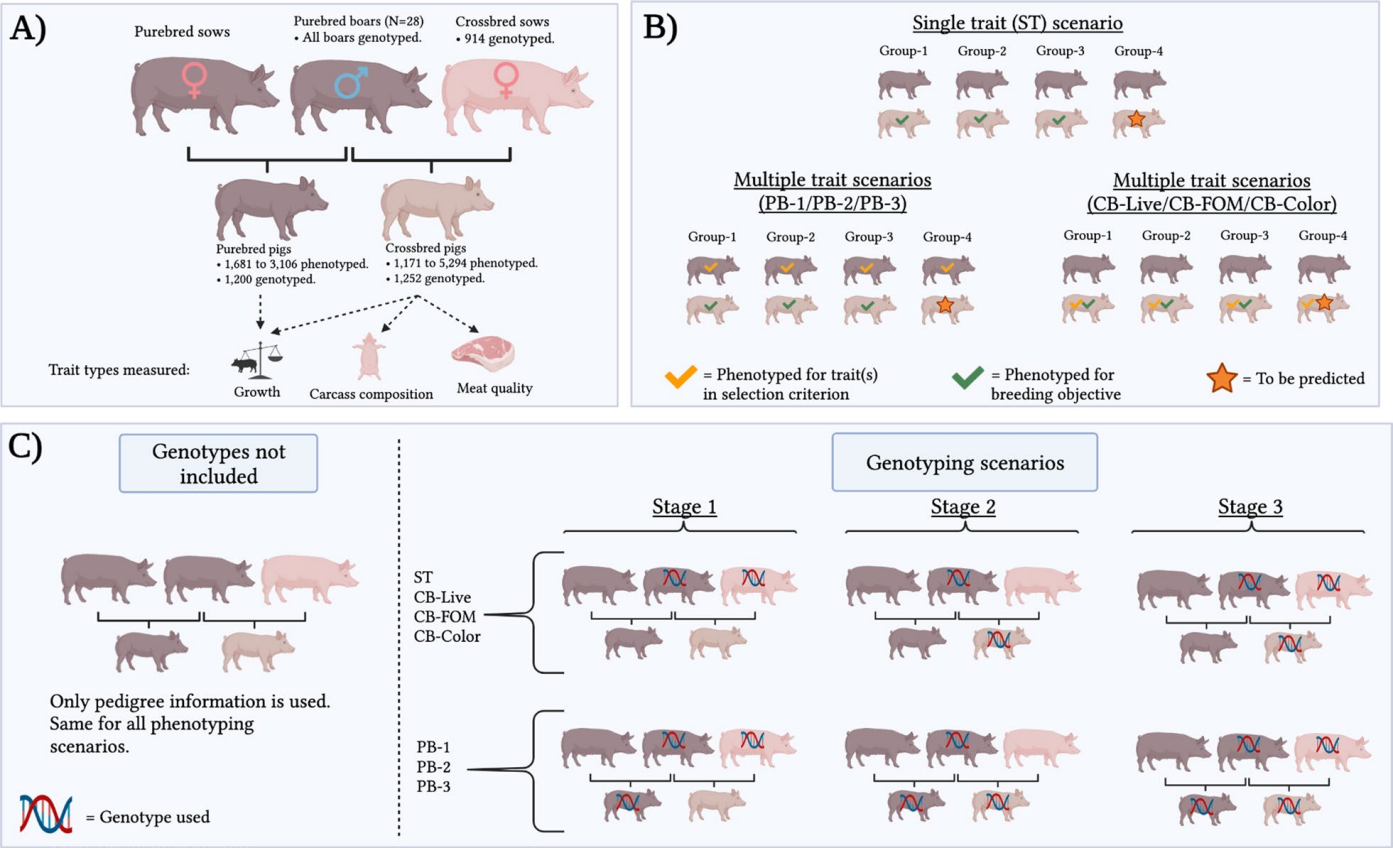
Animals and information included in the genetic prediction scenarios. **a** Shows the relationship between purebred and crossbred pigs, as well as the number of phenotypes/genotypes collected for purebred and crossbred parents and offspring. **b** Shows the source of phenotypic information used for the genomic prediction analysis for each phenotyping scenario, with the diagram showing an example where crossbred offspring of group-4 are the animals to be predicted. **c** Shows the sources of genomic information for each genotyping scenario. The figure was created with BioRender.com

Different genotyping strategies were also evaluated. A first scenario, defined as 'No Genotyping', was used as a baseline, and predictions were obtained using pedigree relationships. In the case of ST, CB-Live, CB-FOM, and CB-Color, the genotyping scenarios 'Stage-1' included genotypes for the 28 PB sires and the 914 dams of CB individuals; 'Stage-2' included genotypes for the 1252 CB individuals and their 28 sires; and 'Stage-3' included genotypes for the sires, dams, and CB individuals. When PB phenotypes were included, the genotypes for the 1200 PB individuals were added to each of the respective genotyping scenarios. A diagram of the information used for each phenotyping/genotyping scenario is in Fig. 1.

For the genomic prediction scenarios, the same models (1) and (2) were used for the cross-validation, using BLUPF90 (v. 1.47) from the BLUPF90 family of programs [25, 26] to obtain estimated breeding values. When genotypes were included, the pedigree-derived relationship matrix $\mathbf{A}$ was replaced with the blended pedigree-genomic relationship matrix $\mathbf{H}$ [29, 30]. The genomic relationship matrix ($\mathbf{G}\mathbf{R}\mathbf{M}$**)** was calculated as described by Lourenco et al. [31], accounting for the different allele frequencies in the populations [32, 33]. A maximum of three populations were considered, defined as (1) the CB individuals, (2) the dams of the CB individuals, and (3) the PB individuals together with the 28 PB sires. We constructed a second genomic relationship matrix that included all individuals and used the average allele frequencies across the three populations to perform eigenvalue decomposition and investigate how the populations were structured.

## Results

### Estimates of genetic parameters

Heritability estimates for crossbred and purebred traits using single-trait models are in Table 1. For crossbred traits, heritability estimates were low, ranging from 0.04 to 0.35, and only four traits, cIMF (0.35), cCBF (0.28), cLBF (0.23), and cSCOL (0.22) had a heritability estimate higher than 0.2. Heritability estimates for the purebred traits were also low, with estimates ranging from 0.11 for pIMF to 0.32 for pLBF.

Estimates of genetic correlations among purebred traits (Table 3) were positive and ranged from 0.29 to 0.97. Purebred live daily gain traits (pLDG1 and pLDG2) were genetically highly correlated (0.72) and estimates of genetic correlations of pLDG1 and pLDG2 with the purebred ultrasound growth traits (pIMF, pLBF, pLLD, and pLLA) ranged from low (0.30) to high (0.79) values. Estimates of genetic correlations between the purebred ultrasound growth traits ranged from 0.29 between pIMF and pLLA to 0.97 between pLLA and pLLD.

**Table 3:** Estimates of genetic correlations among purebred traits

	pIMF	pLBF	pLLD	pLLA	pLDG1
pLBF	0.83 (0.71, 0.94)				
pLLD	0.38 (0.04, 0.68)	0.49 (0.24, 0.68)			
pLLA	0.29 (− 0.05, 0.59)	0.39 (0.13, 0.64)	0.97 (0.94, 1.00)		
pLDG1	0.30 (− 0.02, 0.58)	0.44 (0.18, 0.72)	0.55 (0.34, 0.73)	0.63 (0.43, 0.78)	
pLDG2	0.51 (0.25, 0.72)	0.79 (0.67, 0.89)	0.69 (0.53, 0.83)	0.70 (0.55, 0.86)	0.72 (0.54, 0.85)

Trait definitions are in Table 1

95% highest posterior density interval for genetic correlation estimates are shown in parenthesis

**Fig. 2:**
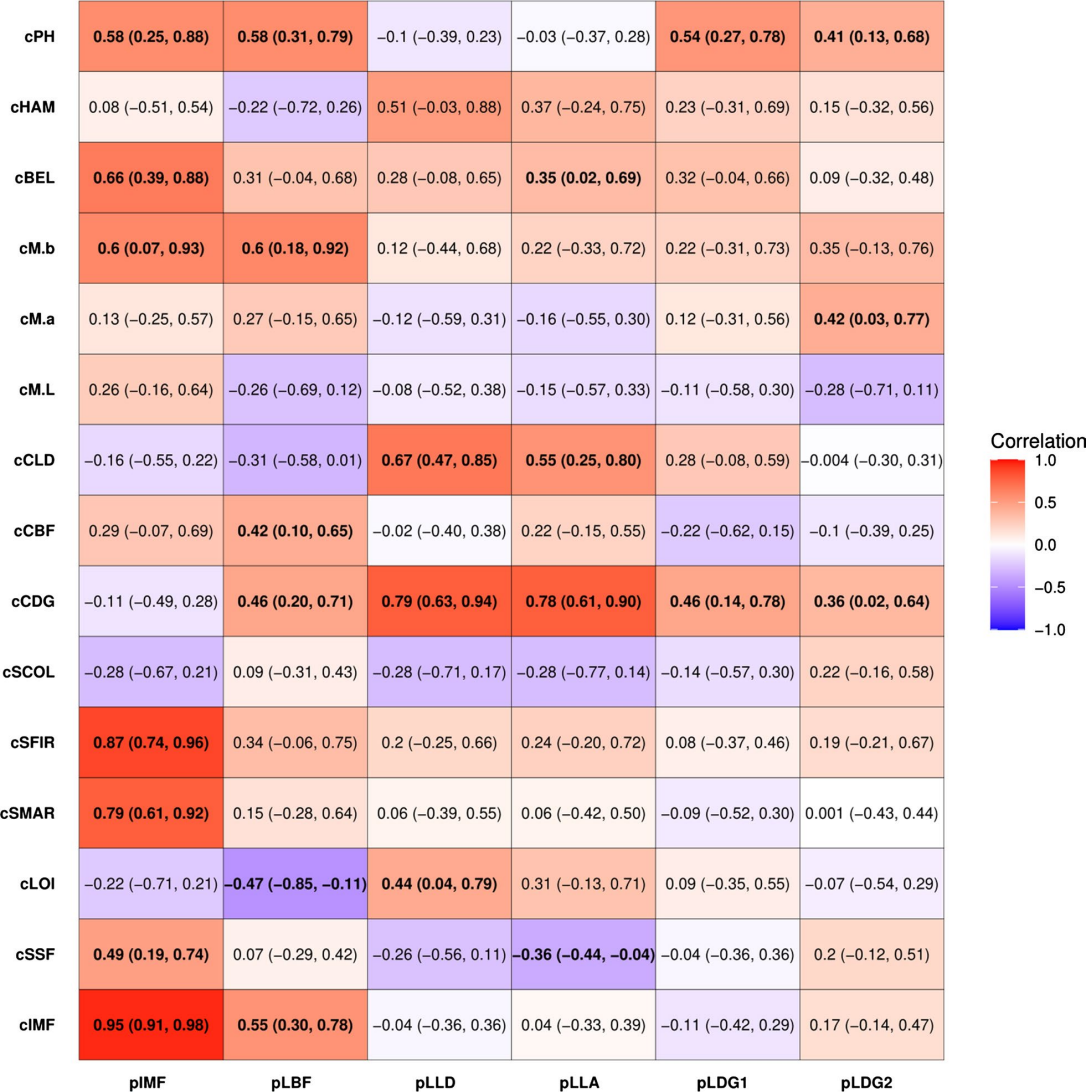
Estimates of genetic correlations between crossbred and purebred traits. The plot shows the strength and direction of the genetic correlations between purebred and crossbred traits. Darker colors indicate a stronger correlation, while red and purple indicate a positive and negative correlation, respectively

Significant (confidence interval did not encompass 0) estimates of genetic correlation of purebred live daily gain with crossbred traits ranged from low to moderate and were all positive, with the highest estimate equal to 0.54 between pLDG1 and cPH. Estimates of genetic correlations of purebred ultrasound growth traits with crossbred traits ranged from low to high and included positive and negative estimates. Both pLLA and pLLD had high and positive genetic correlation estimates with cCDG (0.78 and 0.79, respectively) and moderate to high and positive estimates with cCLD (0.67 and 0.55, respectively). Purebred backfat (pLBF) had the strongest genetic correlation estimates with crossbred meat quality traits, including cM.b (0.60), cPH (0.58), and cIMF (0.55). Purebred intramuscular fat also had the strongest genetic correlation estimates with crossbred meat quality traits, including high and positive estimates with cIMF (0.95), cSFIR (0.87), and cSMAR (0.79), and moderately high estimates with cBEL (0.66), cPH (0.58), and cSSF (0.49). A heatmap of the genetic correlation estimates between crossbred and purebred traits is in Fig. 2.

**Fig. 3:**
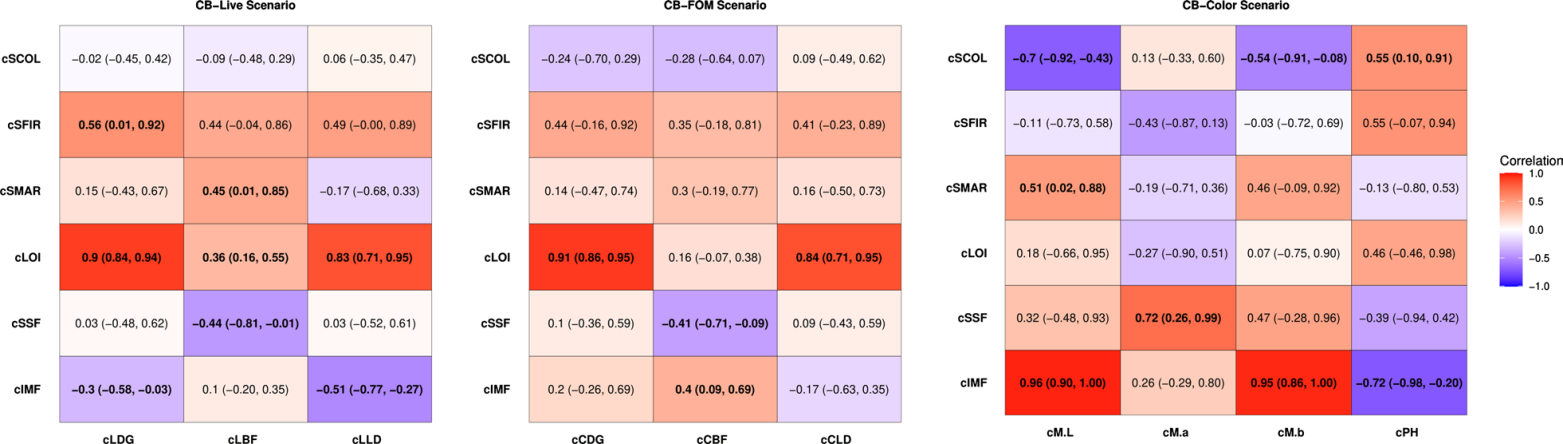
Estimates of genetic correlations between the crossbred traits in the breeding objective in selection index for scenarios CB-Live, CB-FOM, and CB-Color. The plot shows the strength and direction of the genetic correlations between the breeding objectives and crossbred traits in the selection criteria. Darker colors indicate a stronger

The crossbred live growth traits (in CB-Live) had low to moderate genetic correlation estimates in both directions with the meat quality breeding objective traits, ranging from − 0.51 between cLLD and cIMF to 0.56 between cLDG and cSFIR. Estimates of genetic correlations of cLOI with cLDG and of cLOI with cLLD were high and positive, at 0.90 and 0.83, respectively. Estimates of genetic correlations of the carcass traits in the CB-FOM scenario with the meat quality breeding objective traits were mostly non-significant, except the estimates for cCBF, which showed correlations of − 0.41 and 0.40 with cSSF and cIMF, respectively. Two traits, cCLD, and cCDG had high and positive estimated genetic correlations with the sole carcass composition trait in this analysis, cLOI (0.84 and 0.91, respectively). The color and pH traits in the CB-Color scenario had moderate to high genetic correlation estimates in both directions with the meat quality breeding objective traits, ranging from − 0.72 to 0.96. The cIMF trait had strong and positive genetic correlation estimates with two Minolta color traits, cM.L (0.96) and cM.b (0.95), and a strong and negative genetic correlation estimate with cPH (− 0.72). Other traits had strong genetic correlation estimates, i.e. cSSF with cM.a (0.72) and cSCOL with cM.L (− 0.70). A heatmap of estimates of genetic correlations among crossbred traits that served as breeding objectives for the selection index and traits that were included in the crossbred selection criterion scenarios is in Fig. 3.

### Population stratification and genomic prediction

**Fig. 4:**
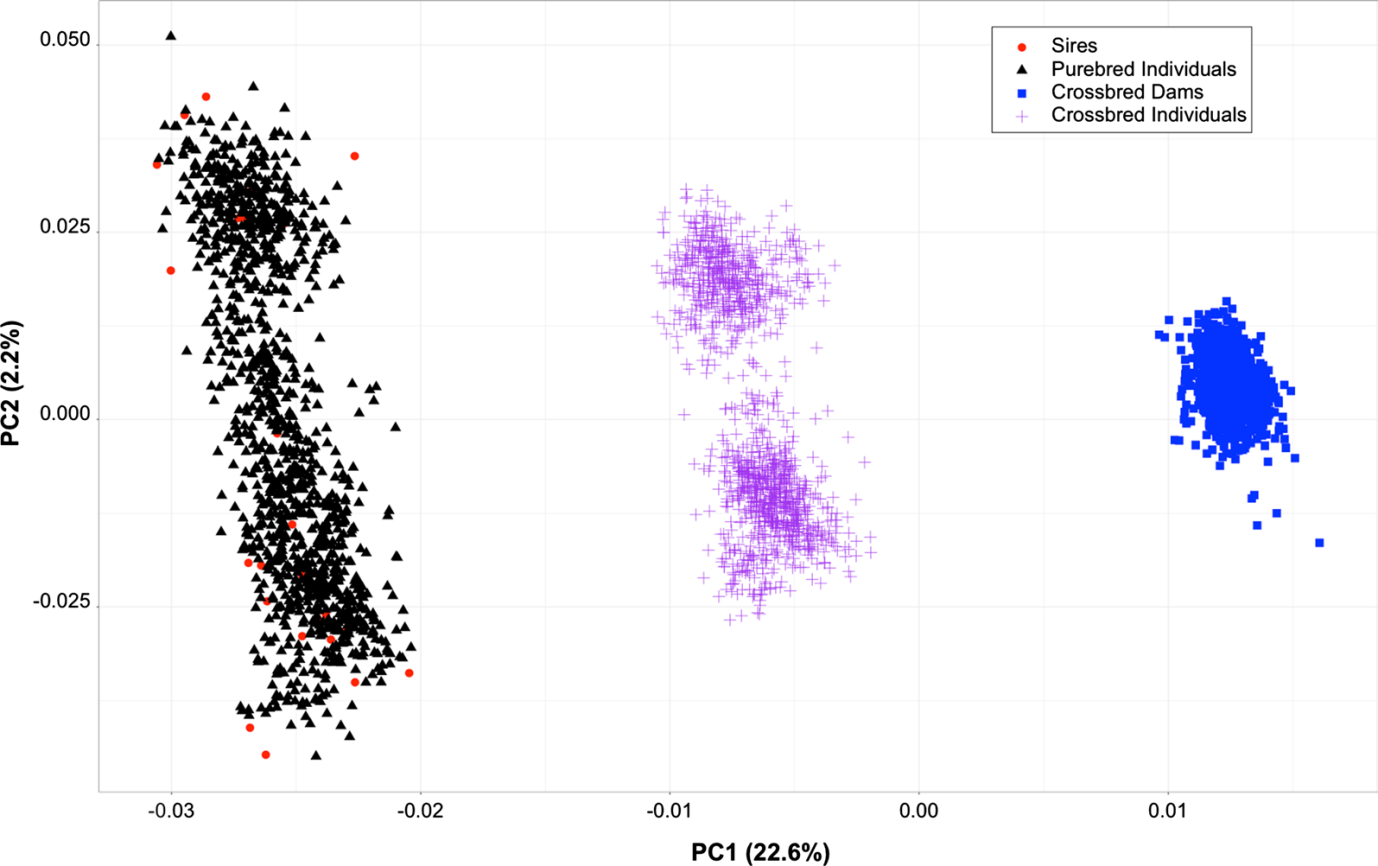
First two principal components for variation in the genomic relationship matrix

Population stratification is reported in Fig. 4, in which the three groups (crossbred individuals, crossbred dams, purebred individuals) are shown and the sires from the purebred population are highlighted. The plots show a clear stratification over the first principal component (eigenvector), which absorbed 22.6% of the genomic variance. The crossbred individuals appear to be equidistant from their crossbred dams and the purebred paternal half-sibs. The second principal component explained only 2.2% of the genomic variance without showing a strong stratification. The 28 sires overlapped with the purebred population, covering most of the variation for both principal components.

**Fig. 5:**
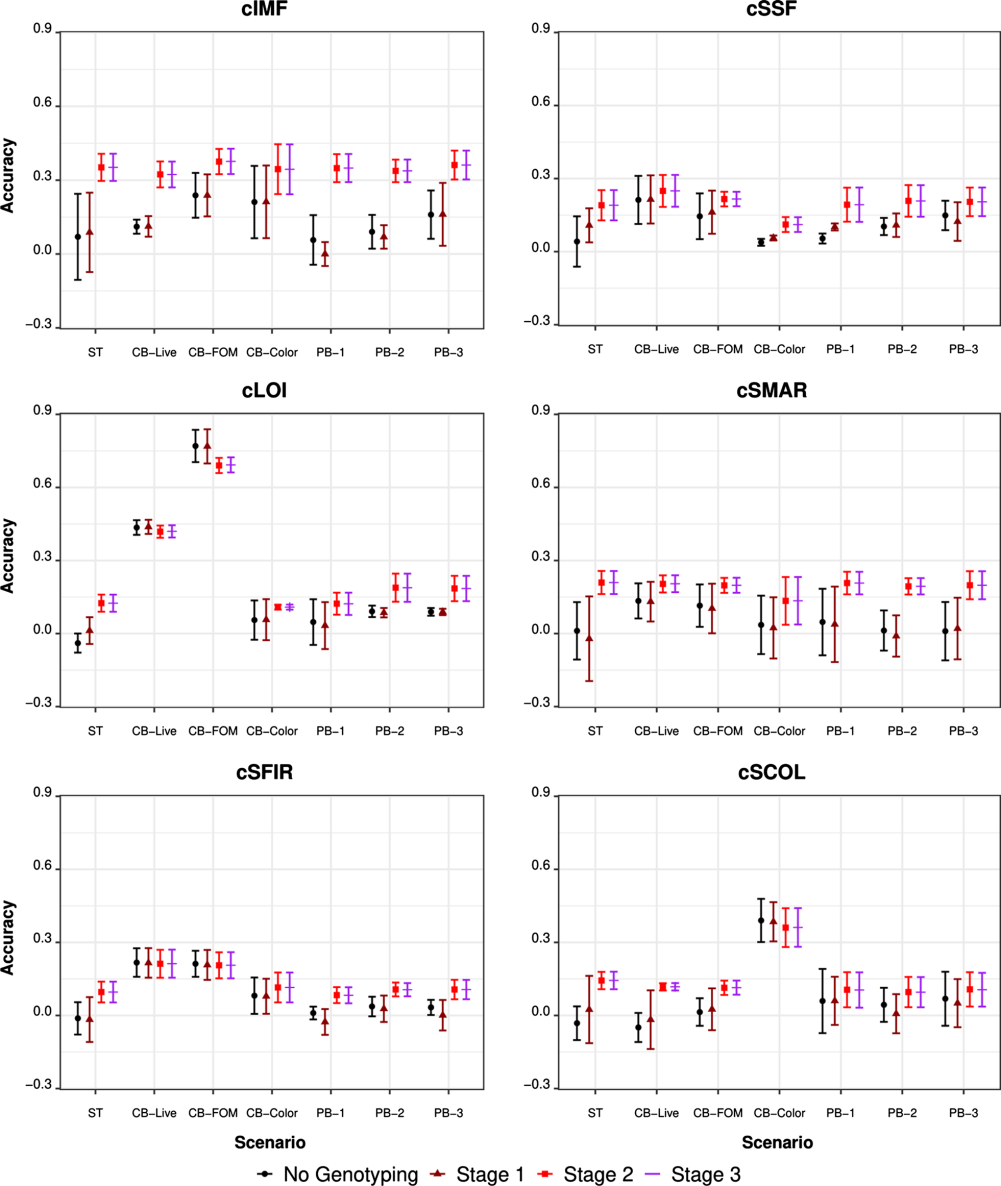
Accuracy of (genomic) prediction using various genotyping strategies in the crossbred phenotyping scenarios. Each panel represents a breeding objective. The x-axis shows the phenotyping scenario (ST/CB-Live/CB-FOM/CB-Color/PB-1/PB-2/PB-3), and the y-axis shows the accuracy obtained. Genotyping scenarios are represented by shape/color combinations: black circle (no genotyping), brown triangle (Stage-1), red square (Stage-2), and purple line (Stage-3). For the definitions of the phenotyping scenarios, please refer to the “Indirect selection for carcass traits using purebred growth phenotypes” and “Less expensive crossbred phenotypes for the selection of meat quality” sections. For the definitions of the genotyping scenarios, please refer to the “Prediction of crossbred traits” section

**Fig. 6:**
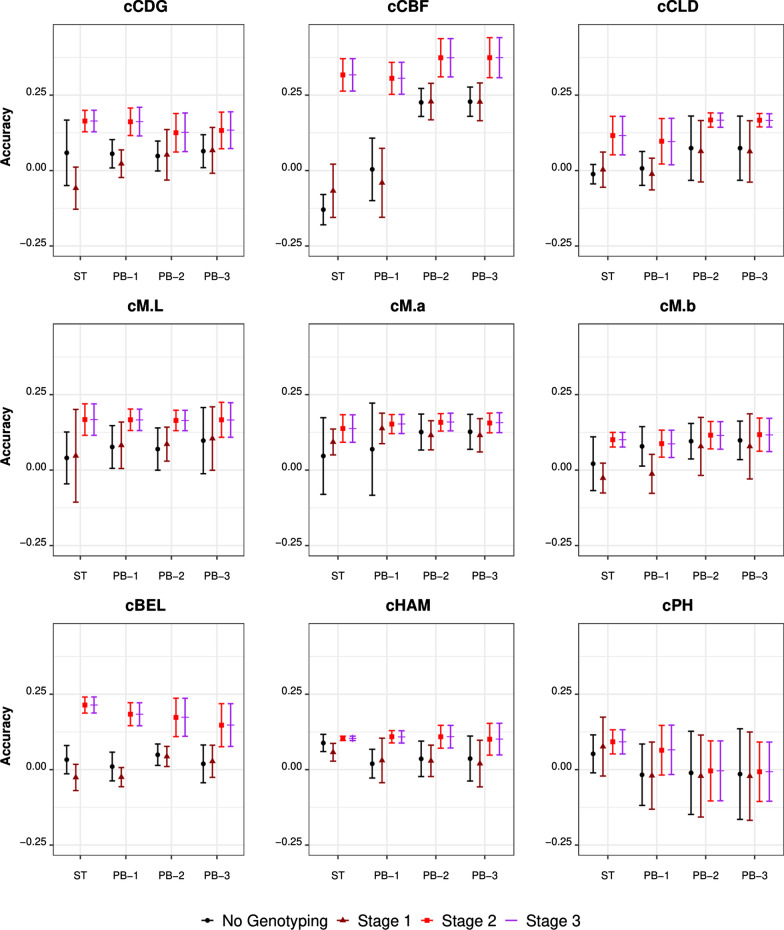
Accuracy of (genomic) prediction using various genotyping strategies in the purebred phenotyping scenarios. Each panel represents a breeding objective. The x-axis shows the phenotyping scenario (ST/PB-1/PB-2/PB-3), and the y-axis shows the accuracy obtained. Genotyping strategies are represented by shape/color combinations: black circle (no genotyping), brown triangle (Stage-1), red square (Stage-2), and purple line (Stage-3). For the definitions of the phenotyping scenarios, please refer to the “Indirect selection for carcass traits using purebred growth phenotypes” and “Less expensive crossbred phenotypes for the selection of meat quality” sections. For the definitions of the genotyping scenarios, please refer to the “Prediction of crossbred traits” section

The accuracies of prediction (mean and standard deviation across the four folds) for the breeding objective traits cIMF, cSSF, cSMAR, cSFIR, cSCOL, and cLOI for the seven phenotyping scenarios (ST, CB-Live, CB-FOM, CB-Color, PB-1, PB-2, and PB-3) and the four genotyping scenarios (‘No genotyping' and Stage-1 through Stage-3) are presented in Fig. 5. For the remaining crossbred breeding objectives, cCDG, cCBF, cCLD, cM.L, CM.a, cM.b, cBEL, cHAM, and cPH accuracies of prediction with four of the phenotyping scenarios (ST, PB-1, PB-2, and PB-3) are presented in Fig. 6.

Phenotyping scenarios had a substantial impact on the accuracy of predictions for cLOI and cSCOL and had a moderate impact on cSFIR, cCBF, and cCLD. For cLOI, the CB-FOM and CB-Live phenotyping strategies resulted in the highest accuracies, with estimates ranging from 0.69 to 0.77 and from 0.42 to 0.44, respectively, across the four genotyping strategies. For cSCOL, the highest prediction accuracy was obtained for the CB-Color phenotyping scenario, with estimates ranging from 0.36 to 0.39, while accuracy was never higher than 0.15 for the other scenarios. For cSFIR, the CB-Live and CB-FOM scenarios had a moderate advantage over the different phenotyping scenarios, with accuracies around 0.20, compared to less than 0.15 for the other scenarios. For cCBF, prediction accuracies were higher for the PB-2 and PB-3 than for the PB-1 and ST scenarios; this was especially noticeable for genotyping strategies that did not include the crossbred individuals (No genotyping and Stage 1). A similar trend was observed for cCLD. Leaving aside the genotyping strategy with commercial crossbred individuals, prediction accuracies for cCBF were zero for the ST and PB-1 phenotyping scenarios, compared to ~ 0.20 for the PB-2 and PB-3 scenarios. We did not observe substantial effects of the phenotyping strategy for the remaining traits, with prediction accuracies reaching the same level for the crossbred and purebred trait phenotyping scenarios as for the single trait strategy (ST) across all four genotyping strategies.

When the genotyping strategy was changed from Stage-1 to Stage-2 or Stage-3, we observed a relevant increase in prediction accuracy for most of the fat-related traits, such as cIMF, cCBF, and cBEL. This was also observed for cCDG in some phenotyping scenarios. The genotyping strategies in which crossbred individuals had genotypes resulted in estimates of prediction accuracy that were, in most cases, higher than for the other scenarios. For cIMF, prediction accuracies for the Stage-2 and Stage-3 genotyping strategies were greater than 0.30 for all phenotyping scenarios, while it never surpassed that value for the other genotyping scenarios. For cCBF, cBEL, and cCDG, prediction accuracies increased to values greater than 0.25, 0.13, and 0.12, respectively, for the Stage-2 and Stage-3 genotyping strategies.

## Discussion

The relative importance of meat quality and carcass composition traits in pork production, coupled with the high cost of phenotyping, has accelerated the need for effective strategies for genetic improvement of crossbred performance for these traits. In this study, we investigated the usefulness of multi-trait (genomic) selection for meat and carcass quality in commercial crossbred individuals.

### Estimation of genetic parameters

First, we estimated the genetic parameters of routinely measured traits in crossbred and purebred pigs. Although available, we did not use genomic data to estimate variance components in this study because, for some traits, all the phenotyped individuals were genotyped (e.g., cIMF, cPH), whereas for other traits only some of the phenotyped individuals were genotyped (e.g., cCLD, cLBF), which explains the difference as compared to our previous work on the same data [11]. Since genetic correlation estimates depend on genomic data and on how this information is used [9, 33], the partial availability of genomic data could have hampered the interpretation of the results.

#### Heritability

Heritability estimates for meat quality traits measured on commercial crossbred animals were low and mainly within the range of previous estimates reported in similar crossbred populations [11, 34]. Khanal et al. [11] reported low heritability estimates for Minolta color, subjective score, and pH traits, ranging from 0.08 to 0.27. Our estimates for the heritability of color traits (cM.L, cM.a, cM.b) and cSSF were notably lower than those reported by Miar et al. [34], which ranged from 0.20 to 0.39. For carcass Fat-O-Meater traits, heritability estimates were lower than those previously reported for purebred [35] and crossbred [11] pigs. Our estimate of the heritability of cCDG was much lower than the estimates reported by Khanal et al. [11] (0.42 to 0.44), which might be due to differences in trait modeling and the use of genomic information in the estimation. Our estimates of the heritability of crossbred growth traits measured at market weight (cLBF, cLLD, and cLDG) were lower than those reported by Khanal et al. [11]. For primal cuts weight traits (cLOI, cBEL, and cHAM), our heritability estimates were similar to those previously reported for purebred and crossbred pig populations [11, 36].

For the traits recorded on purebred individuals, estimates of the heritability of ultrasound growth measures were low and similar to those previously reported in crossbred animals (0.26 to 0.45) [12]. Although our heritability estimates for average daily gain were in line with the low estimates reported by Miar et al. [12] and Willson et al. [36], they were significantly lower than the estimate of 0.67 reported in Duroc pigs by Cabling et al. [37].

#### Purebred–crossbred genetic correlations

Previous estimates of the purebred–crossbred correlation for the same trait in pigs range from 0.47 to 0.99 for growth [5–8], from − 0.10 to 0.96 for carcass composition [5–8], and from − 0.22 to 1.00 for meat quality [8]. In our study, genetic correlations between purebred growth and crossbred carcass measures were obtained to elucidate their relationship and understand to what extent high-value carcass traits in crossbred pigs would respond to indirect selection using routinely measured purebred traits. Estimates of genetic correlations between purebred average daily gain and crossbred carcass traits were, for the most part, non-significant, except for the correlations with cM.a, cPH, and cCDG. A moderate genetic correlation estimate of 0.56 between cCDG and purebred average daily gain (from birth to finishing) was previously reported [6]. Ultrasound growth measures of backfat, loin depth, loin area, and intramuscular fat in purebreds were found to have significant genetic correlation estimates with cIMF, cSSF, cM.b, cPH, cSMAR, and/or cSFIR, which were mostly moderate to high and positive. While the lack of research on the genetic correlations between purebred growth and crossbred meat quality traits precludes any direct comparisons with the majority of our findings, our estimates of the genetic correlation between purebred intramuscular fat and crossbred meat quality traits (specifically cIMF, cSMAR, and cPH) are slightly larger than those reported by Esfandyari et al. [8]. Another study that was conducted on crossbred pigs only [12], found mostly weak and non-significant genetic correlations between ultrasound measures of backfat, loin depth, and intramuscular fat and meat quality, with the exception of correlations between pH and loin depth (− 0.49), pH and intramuscular fat (0.73), and subjective marbling and intramuscular fat (0.59). For the other crossbred carcass traits, significant estimates of genetic correlations with purebred ultrasound growth traits were moderate to high, with the strongest correlations being with cCLD, cCDG, and cBEL. Evidence of a genetic correlation between ultrasound growth and carcass composition traits has already been reported in crossbreds [12] and between purebred and crossbreds [6]. Our estimates for the genetic correlation between purebred and crossbred measures of fat and loin depth showed a moderate to strong and positive relationship, which is in line with previous estimates [6, 8, 12]. We did not observe a clear pattern for the genetic correlation estimates between ultrasound growth and primal weight traits. Miar et al. [12] found a low and mostly non-significant genetic relationship between these traits.

#### Genetic correlations between traits measured on crossbreds

Due to the varying cost of collecting carcass measures on commercial crossbred animals, phenotyping the crossbred offspring of purebred selection candidates for cheap and routinely measured crossbred traits is a possibility worth exploring. The three crossbred selection criterion scenarios evaluated here represent potential avenues of phenotype collection. However, their effectiveness in selection will largely depend on the genetic correlation between these traits and the traits of interest (breeding objective traits). Genetic correlation estimates between meat quality and growth traits were mostly not significant, which suggests a lack of a shared genetic architecture. An exception to this was the moderate correlations of cCBF with both cIMF and cSSF. Khanal et al. [11] found similar results in two separate populations, with cCDG and cCLD having a genetic correlation close to zero and cCBF having low to moderate correlations with meat quality traits. In addition, these results are also consistent with the low and positive relationships between carcass and meat quality traits reported by Miar et al. [34].

Not surprisingly, the meat quality traits in the CB-Color selection criterion scenario had the strongest genetic correlations with the meat quality breeding objectives. Other studies have also found significant and moderate to strong genetic correlations between meat quality traits, especially intramuscular fat, subjective color, and Minolta color traits [11, 34].

### Effect of genotyping and phenotyping strategy on genetic progress and prediction accuracy

In this study, we used two methods to assess the value of using purebred and crossbred information (genomic and phenotypic) to select for expensive-to-record traits measured on crossbred individuals. The estimation of variance components set the basis for the comparison of the different scenarios. A cross-validation was also used to test the ability of each model to predict future crossbred performance. We chose to predict crossbred performance for several reasons, such as (1) CB performance is the breeding goal, (2) using real data, we did not know the true breeding value of the PB selection candidates, (3) ranking the CB individuals appropriately means ranking the paternal half-sib families among each other as well as the individuals within the family. As for (1), we opted for the prediction of the trait itself because it is the performance of the CB individual that determines the remuneration to the farmer, while we adjusted the phenotype for the systematic effects that are controllable in the breeding scheme. As for (2), the accuracy of PB (G)EBV for CB traits was lower than ‘1’, and this would not have provided the proper ground for comparison of the models. For example, predicting the PB EBV or GEBV would have been a substantially different comparison and could have underestimated or overestimated the value of using genomic information. Finally, for (3), as the model was ranking appropriately the ~ 300 CB individuals, it was also (i) ranking the seven paternal half-sib families, which were sired by the seven PB Duroc sires while (ii) ranking individuals within the family. The feature of (i) challenges the model in identifying the PB individuals based on their CB progeny performance, while the feature of (ii) allows the evaluation of the impact of Mendelian sampling within family. Overall, we found that the prediction of CB individuals’ performance provides the best ground for comparison while providing the most precise picture for comparing different genotyping and phenotyping strategies.

The estimates of genetic variances and covariances may be not completely exhaustive, since a cross-validation also acknowledges that the genetic (co)variances cannot completely capture the genetic architecture of a trait, and thus cannot reveal how the information flows across the populations used in this study. Moreover, cross-validation was also used to assess the impact of genotyping strategy on the (multi-trait) prediction of the crossbred traits.

#### Selection for crossbred traits using information from purebreds in the absence of genomic information

The PB-1 scenario mimicked the selection of purebred growth traits (pLDG1 and pLDG2) to improve the crossbred traits. The cross-validation did not show an advantage of this approach over direct phenotyping of the breeding objective. The PB-2 scenario, which included purebred ultrasound traits (backfat and muscle), showed an improvement over direct selection for cBF, cLD, and cLOI. The cross-validation did not show a comparable outcome for cIMF, cSSF, meat color traits (cM.b, SCOL) and cHAM. The PB-3 scenario (which included intramuscular fat as obtained by ultrasound on the purebred individuals) mildly increased the accuracy for cIMF but not for cHAM, cBEL, cSFIR, and cM.L.

#### Selection for crossbred traits using cheaper-to-measure traits in the absence of genomic information

We investigated three phenotyping scenarios that aimed at improving crossbred traits that are expensive to measure using traits that could be more easily recorded on the same crossbred individuals. We chose measures of meat quality (marbling, color, and tenderness), both objective and subjective, as well as a measure of carcass quality (loin muscle weight), all requiring carcass dissection and wet-lab analyses for their assessment.

The CB-Live scenario included measures that can be collected on live crossbred animals using a weight scale and an ultrasound probe. The cross-validation showed a clear advantage for using this scenario on cLOI, cSFIR and cSSF under this scenario. The CB-FOM scenario included traits that can be collected post-mortem without dissecting the carcass. This scenario showed a further improvement for cLOI of 75% in prediction accuracy. The CB-Color phenotyping scenario included objective meat color measures as predictor traits, which do not need dissection of the whole carcass to be recorded but require that the loin muscle is exposed, which increases labor requirements. The cSCOL trait showed a substantial increase in prediction accuracy under cross-validation, reaching the only non-null prediction accuracy under this scenario. Conversely, cIMF showed no particular advantage under this scenario.

#### On the value of using crossbred/purebred genomic information in prediction

We compared the increase in predictive ability that can be achieved by including genomic information, either recorded on the sires, dams, or purebred/crossbred progeny. The Stage 1 genotyping scenario included the genotypes of the 28 sires together with the genotypes of the crossbred dams. In Stage 2, the genotypes of the crossbred individuals were included, whereas in Stage 3, genotypes of the purebred sires and dams, and of the crossbred individuals were included. Stages 2 and 3 aimed at mimicking scenarios where the crossbred individuals are effectively included in the training population. The use of genotypes of crossbred individuals is non-conventional, as they are not used for breeding, however they could add relevant information to the training population, especially when the economically important traits are recorded on the crossbreds. When purebred phenotypic records were included (phenotyping scenarios PB-1, PB-2, and PB-3), the genotypes of purebred individuals were also included for all genotyping scenarios.

The results showed a clear picture of the relevance of genomic information from each group (sires, dams, and purebred/crossbred progeny). The inclusion of genotypes of the sires and dams was irrelevant in terms of predictive ability, under all the phenotyping scenarios and for all traits. A substantial advantage was found when including genotypes of the crossbred individuals, although this varied across traits and phenotyping scenarios. The traits that benefitted the most from including genotypes on crossbreds were the fat-related traits: cIMF, cBEL, and cCBF. The advantage for these traits was strong and consistent across the phenotyping scenarios and showed that inclusion of genotypes of crossbreds leads to an improvement in multi-trait prediction accuracies under any phenotyping scenario. These were followed by growth and meat quality traits, such as cCLD, cSCOL, cCDG, and cM.b.

In this study, we tested the value of purebred genotypes only when their phenotypes were included, so the contribution of these two components could not be disentangled. However, it is evident that inclusion of genotypes/phenotypes on purebreds seldom improved the models' predictive ability. Comparison of the predicting performance within the PB-1/2/3 phenotyping scenarios showed that using genotypes from sires and dams (Stage-1) did not improve the accuracy of predicting crossbred performance and that the predictive ability increased only when genotypes on crossbreds was included, even in the presence of genotypes on purebreds.

The results suggest a decisive advantage of including crossbred genomic information into the training population when performing genomic selection on traits recorded on the crossbred individuals if the validation is also done on those individuals. The same conclusions were drawn by Esfandyari et al. [38] using simulated data. Lourenco et al. [31] found an increase in prediction accuracy of 39% for crossbreds when their genotypes were included in the evaluation, compared to a scenario where only the genotypes of the purebred parents were used. Based on simulations, van Grevenhof and van der Werf [15] showed that crossbred animals should be included in the reference population when the objective is to select for crossbred performance and the correlation between purebred and crossbred performances is lower than 0.7. At the same time, Wientjes et al. [39] found a lower prediction accuracy for crossbred performance using a purebred reference population when the purebred–crossbred correlation was 0.5.

#### Investing in genotyping vs. phenotyping

We compared genotyping and phenotyping scenarios using real data recorded on 15 traits. We used cross-validation to compare the phenotyping strategies. The overall results suggest that the advantage of genotyping vs. phenotyping depends on the target trait(s). Some traits saw a sizeable benefit from genotyping the crossbred individuals that were phenotyped. These traits are those that are routinely considered in genetic evaluation, such as cCDG, cCBF, and cCLD, but also cBEL and, ultimately, cIMF. Genomic selection for these traits could be performed without including additional traits in the selection index, provided that the reference population is constantly updated. Some other traits, i.e., cLOI, cSFIR, and cSCOL did not benefit from the inclusion of genomic information but did benefit from the inclusion of correlated traits in the multi-trait model. In this case, the crossbred individuals to be predicted were phenotyped for the predictor traits but had no information on the breeding goal trait. In particular, for cSCOL a minimal advantage was obtained from the inclusion of genomic information.

Our results make it difficult to infer the reasons for a stronger advantage of genotyping vs. multi-trait phenotyping or vice versa. Overall, the traits for which there was an advantage of genotyping or multi-trait phenotyping showed comparable values of heritability and genetic correlations with the other traits. The factors that influence the advantage of including genomic information could be the presence of a different number of quantitative trait loci that determine the trait, but also the number of effective chromosome segments and the linkage disequilibrium between the marker loci and the loci that determine the traits [40]. In turn, the number of records added to the model could influence the advantage of adding phenotypic information. Beyond these known factors, real data could hide additional factors that determine the advantage of one component versus the others.

We also acknowledge the need to study the use of methods that incorporate marker information based on the breed of origin of the allele, which has produced promising results in some studies [41–43]. However, the lack of genomic information on the maternal lines did not allow us to determine the breed of origin of the alleles.

## Conclusions

This study investigated the use of different phenotyping and genotyping strategies to maximize predictive ability for crossbred performance using real data. We found sizeable genetic correlations between purebred growth and crossbred carcass, color, and meat quality traits. The cross-validation results showed that the accuracy of phenotype prediction differed between genotyping and phenotyping strategies. In addition, we demonstrated that availability of crossbred genotypes improved prediction accuracy across most of the phenotyping scenarios. We recommend the inclusion of crossbred individuals in the reference population when the breeding goal traits are recorded on these individuals. Our findings provide evidence of a genetic relationship between traits measured in crossbred and purebreds that could be exploited under several phenotyping and genotyping scenarios to improve crossbred performance.

## Data Availability

Not applicable.
